# Supporting a Role for the GTPase Rab7 in Prostate Cancer Progression

**DOI:** 10.1371/journal.pone.0087882

**Published:** 2014-02-05

**Authors:** Joshua J. Steffan, Samantha S. Dykes, David T. Coleman, Lisa K. Adams, Donna Rogers, Jennifer L. Carroll, B. Jill Williams, James A. Cardelli

**Affiliations:** 1 Department of Microbiology and Immunology, Louisiana State University Health Sciences Center, Shreveport, Louisiana, United States of America; 2 Feist-Weiller Cancer Center, Louisiana State University Health Sciences Center, Shreveport, Louisiana, United States of America; 3 Department of Biochemistry and Molecular Biology, Louisiana State University Health Sciences Center, Shreveport, Louisiana, United States of America; 4 Department of Urology, Louisiana State University Health Sciences Center, Shreveport, Louisiana, United States of America; 5 Department of Natural Science, Dickinson State University, Dickinson, North Dakota, United States of America; Queensland University of Technology, Australia

## Abstract

Invasion and subsequent metastasis is the major cause of death from most cancers including prostate cancer. Herein we report on the potential tumor suppressive properties of Rab7, a GTPase that regulates trafficking of lysosomes. The movement of lysosomes to the cell surface in response to environmental cues increases the secretion of proteinases and cell invasion. We determined that Troglitazone and other members of the Thiazolidinedione family inhibit cell-surface directed lysosome trafficking and cathepsin B secretion through a Rab7-dependent mechanism. Moreover, Rab7 shRNA expressing cells were found to be more invasive *in vitro* and *in vivo*. Increased invasiveness was accompanied by elevated expression of the c-Met receptor and prolonged downstream signaling, thereby supporting a role for Rab7 as a mediator of signaling down-regulation. Taken together, these results suggested that Rab7 acts as a negative regulator of prostate tumor growth and invasion, providing further evidence for its potential as a tumor suppressor.

## Introduction

Formation of metastatic foci from an invasive primary tumor is the pivotal event in the process leading to cancer-associated deaths. Mortality rates for late-stage prostate cancer (metastatic disease) have not improved significantly over the past decade; therefore, an understanding of the mechanisms underlying tumor invasion and metastasis, and the development of therapies to prevent tumor progression are urgently needed. The hepatocyte growth factor (HGF)-Met signaling axis is an important regulator of tumor cell invasion and metastasis [1]. Ligand-induced c-Met signaling is known to induce an epithelial-mesenchymal transition (EMT) in which epithelial cells lose cell-cell adhesions and take on a more motile, mesenchymal phenotype [2]. The induction of EMT is thought to enhance tumor cell migration and invasion through basement membranes. In addition to Met signaling, other factors within the tumor microenvironment such as hypoxia, aerobic glycolysis, and acidic extracellular pH (pHe) can increase tumor cell invasion [3], [4].

Tumor invasion often requires the secretion of proteolytic enzymes, although some forms of cell migration/invasion are protease-independen [5], [6]. Although proteinase secretion can occur via the constitutively active secretion pathway, major roles for lysosome-localized proteases have been demonstrated in tumor cell invasion [7], [8]. We have previously demonstrated that acidic pHe and HGF induce the trafficking of lysosomes to the cell surface, resulting in increased cathepsin B secretion [9], [10]. This is significant as altered cathepsin B and D localization is detected in breast and melanoma models in response to Ras transformation or changes in pHe [11], [12]. In previous publications, we have demonstrated that the spatial location of lysosomes in tumor cells is important for cell invasion [10], [13]. When lysosomes are positioned closer to the cell surface, an increase in secreted proteases and cell invasion is detected; whereas, when lysosomes are located nearer the cell nucleus (away from the cell surface; i.e. juxtaposed to the cell nucleus) tumor cells secrete fewer proteinases and are significantly less invasive.

Lysosomes are trafficked throughout cells along microtubules and/or actin filaments utilizing molecular motor proteins such as dyneins, kinesins, and/or myosin family members [14]. In addition, several GTPases such as RhoA, Rab7, Rab27, and Arf family members regulate motor protein activity or recruitment, thus regulating the distribution of lysosomes and other endocytic vesicles [15], [16].

Rab7 is a regulator of intracellular endocytic/membrane trafficking and is involved in many disease states including cancer and several infectious diseases (reviewed in [17]). Rab7, with one of its effectors, RILP (Rab7-interacting lysosomal protein), recruit the dynein-dynactin motor complex to lysosomes facilitating lysosome trafficking along microtubules towards the cell nucleus [18]. Moreover, in addition to its recognized role in vesicle trafficking, Rab7 has recently garnered attention as a regulator of apoptosis in response to growth factor withdrawal [19] and has been proposed to function as a tumor suppressor protein [20].

Inhibitors of sodium-proton exchangers (NHEs) have been shown to be very effective in preventing cell-surface directed lysosome trafficking, thereby decreasing protease secretion and cell invasion [9], [10], [13]. EIPA (5-(*N*-ethyl-*N*-isopropyl)-amiloride) has been shown to prevent acidic pHe and HGF-induced cell surface-directed lysosome trafficking; whereas, members of the thiazolidinedione family of compounds have been shown to prevent acidic pHe-induced cell surface-directed lysosome trafficking. Our previous work determined the compounds troglitazone and EIPA both utilize a Rab7-dependent mechanism to induce juxtanuclear lysosome aggregation (JLA) [13]. Troglitazone is one of several members of the thiazolidinedione (TZD) family of synthetic high-affinity ligands for the transcription factor peroxisome proliferator-activated receptor-γ (PPAR- γ). However, these compounds, including ciglitazone (Cig), rosiglitazone (Rosi; Avandia), and pioglitazone (Pio; Actos), exhibit PPAR- γ independent effects, and our laboratory has demonstrated the potent effect of troglitazone on acidic pHe-induced lysosome distribution to be independent of PPAR- γ but requiring Rab7 (TZDs reviewed in [21]). In fact, it was recently demonstrated that lysosomes traffic to the cell surface in cells expressing Rab7 shRNA (independent of external stimulus) suggesting that Rab7 is a negative regulator of cell surface-directed lysosome trafficking [10]. In addition, Rab7 shRNA expressing cells exhibited increased levels of secreted proteases and were more invasive *in vitro* [10], [13].

Herein, we present several lines of evidence demonstrating that Rab7 may play a suppressive role in tumor growth and progression, suggesting for the first that Rab7 is a putative tumor suppressor *in vivo*. Troglitazone required Rab7 to prevent HGF-induced protease secretion and tumor cell invasion *in vitro.* In addition, Rab7 shRNA expressing cells formed larger tumors *in vivo*, which exhibited increased proliferation, decreased apoptosis, and increased invasive capacity into surrounding tissues. Finally, shRNA mediated reduction of Rab7 led to an increase in c-Met *in vitro* and *in vivo* providing a possible mechanism to account for these changes.

## Materials and Methods

### Ethics Statement

No human tissue was used in this study. All of the animals used in this study received humane care based on guidelines set by the American Veterinary Medical Association (AVMA) as well as in accordance with the Guide for the Care and Use of Laboratory Animals (Institute for Laboratory Animal Research, Washington, DC). All protocols involving live animals are reviewed and approved by the Institutional Animal Care and Use Committee of LSU Health Sciences Center-Shreveport. This set of experiments was conducted under the approved protocol P-07-059. All efforts were made to minimize animal suffering, to reduce the number of animals used, and to utilize alternatives to in vivo techniques, if available.

### Cell Culture

The human prostate cancer cell line DU-145 was purchased from ATCC and maintained in RPMI-1640 (Mediatech) with 10% FBS (Gemini Bio-Products) and 1% Penicillin-Streptomycin (Mediatech). Cells were maintained in a 37°C incubator with 5% CO_2_ and were sub-cultured upon attaining >75% confluence.

### Reagents and Antibodies

Phalloidin, 1∶100 was purchased from Molecular Probes. α-Tubulin antibody, 1∶1000, was purchased from Lab Vision. LAMP-1 antibody (H4A3), 1∶50, was purchased from the Developmental Studies Hybridoma Bank at the University of Iowa, USA. The following antibodies were used for Western bloting: pMet, pAkt, pErk1/2, (1∶1000), cleaved-caspase-3 (1–200) (Cell Signaling Technology, Beverly, MA, USA), Rab7 (1∶1000) (Sigma, St Louis, MO, USA), c-Met (tissue samples 1∶500) (Abcam, Cambridge, MA, USA), c-Met (1∶1000) (Invitrogen, Carlsbad, CA, USA), Ki67 (1∶50) (Thermo Fisher Scientific, Rockford, IL). Fluorophore-conjugated secondary antibodies (1∶100) were purchased from Jackson Immunoresearch Laboratories (Westrgrove, PA, USA). IHC secondary antibodies (1∶200) were purchased from Vector Labs (Burlingame, CA, USA). HGF (Calbiochem, San Diego, CA, USA) was used at 33 ng/ml.

### Extraction of Protein from Frozen Tumor Samples

Frozen tumor samples were first diluted in ice cold RIPA buffer containing Roche protease inhibitors cocktail (Indianapolis, IN, USA), NaF, and NaVO4 at a 1∶5 ratio, mass to volume. Samples were manually homogenized using a mortar and pestle followed by brief sonication, and placed on ice for 20 minutes with periodic vortexing. Samples were then centrifuged at 12,000 g for 10 minutes to remove insoluble debris. Protein concentrations were determined by BCA assay and equal protein was diluted in Laemmli buffer and boiled for 10 minutes.

### Cathepsin B Secretion Assay

Assay was performed as previously described [9], [10]. HGF was added to cultures for 18 hrs at 33 ng/ml.

### Immunofluorescence (I.F.) Staining and Microscopy

I.F. microscopy was performed as previously described [9], [10]. Briefly, cells were fixed with ice cold 4% PFA for 20minutes. Cells were then washed in PBS then incubated with anti-LAMP-1 (H4A3-s Iowa State University Hybridoma Bank) at a 1∶100 dilution in BSP for one hour at room temperature. Cells were then washed, and incubated with Dylight 594 anti mouse (Jackson IR) at a 1∶100 dilution in BSP for one hour at room temperature. Phalloidin was then used to stain the actin cytoskeleton (Phalloidin 488, Molecular Probes) and was incubated for 20 minutes at 1∶200 in BSP at room temperature. Slides were mounted with DAPI containing SlowFade gold reagent (Invitrogen S36938). Cells were imaged using an Olympus BX-50 microscope using MetaMorph software. Images were merged using ImageJ.

### Lysosome Distribution Analysis

Lysosome distribution from the nucleus was measured using the LysoTracker software, a generous gift from Meiyappan Solaiyappan (Johns Hopkins). A total of 25 cells spanning from three independent experiments were analyzed for each data set.

### Western Blot Analysis

Western blot analysis was performed as previously described [9].

### Lentiviral Delivery of Short-hairpin RNA

Short hairpin RNAs (shRNA) directed towards Rab7 (CCGGGCCACAATAGGAGCTGACTTTCTCGAGAAAGTCAGCTCCTATTGTGGCTTTTT )were delivered into DU145 prostate cancer cells (creating stable Rab7 shRNA expressing cells) using Mission Lentiviral Transduction Particles (Sigma) according to manufacturer’s protocol and as previously described [9]. Non Target shRNA expressing cells were generated using the same method with a control vector targeting no known mammalian genes (Sigma shc202V).

### Invasion Assay

Invasion assays were performed as previously described [10], [13]. HGF was added in serum free media to both the top and bottom chambers at a concentration of 33 ng/ml. Results represent the average number of invaded cells in four fields of view per condition from three independent experiments.

### *In vivo* Experiments

Six to 8 week old male SCID/bg mice were injected with 2×10^6^ DU145 prostate tumor cells stably expressing either scrambled (non-target; NT) shRNA or shRNA directed to Rab7 in 100 µl PBS subcutaneously (n = 6 NT shRNA; n = 7 Rab7 shRNA). Tumors became measurable by day 41 post-implantation and were measured with digital calipers twice per week. Tumor volumes were calculated by the equation: Volume = π/6 * Length * Width^2^. Mice were euthanized at 104 days post-implantation and tumors were surgically removed and fixed in 10 percent formaldehyde or frozen. All experiments were performed in accordance with guidelines set by the LSUHSC-S Institutional Animal Care and Use Committee.

### Immunohistochemistry

Immunohistochemistry was performed utilizing standard DAB techniques. Briefly, the formalin fixed tumors were processed and embedded in paraffin. These blocks were then sectioned, deparaffinized, and rehydrated in xylene and graded ethanol washes. Antigens were unmasked with preheated antigen retrieval buffer for 15 min and then cooled. 0.3% H_2_O_2_ in methanol was placed on the slides for 30 min to block endogenous peroxides, followed by a protein (serum) block for 10 min. Primary antibody was then added for 1 hr at the indicated dilution. Biotinylated secondary antibody (1∶200) was then added for 30 min. Biotin binding was increased using the ABC Elite method (Vector Labs) for 30 min and stains were visualized with DAB (Vector Labs) for 7 min. Slides were counterstained with hematoxylin for 2 min.

### Statistical Analysis

GraphPad Software, Prism 3.0 was utilized to perform all statistics. One or Two-Tailed Mann-Whitney T-tests were performed to indicate statistical significance. All graphs show the standard error of the mean (s.e.m.).

## Results

### Troglitazone Prevents HGF-induced Cell Surface-directed Lysosome Trafficking, Cathepsin B Secretion, and Cell Invasion

**Figure 1:**
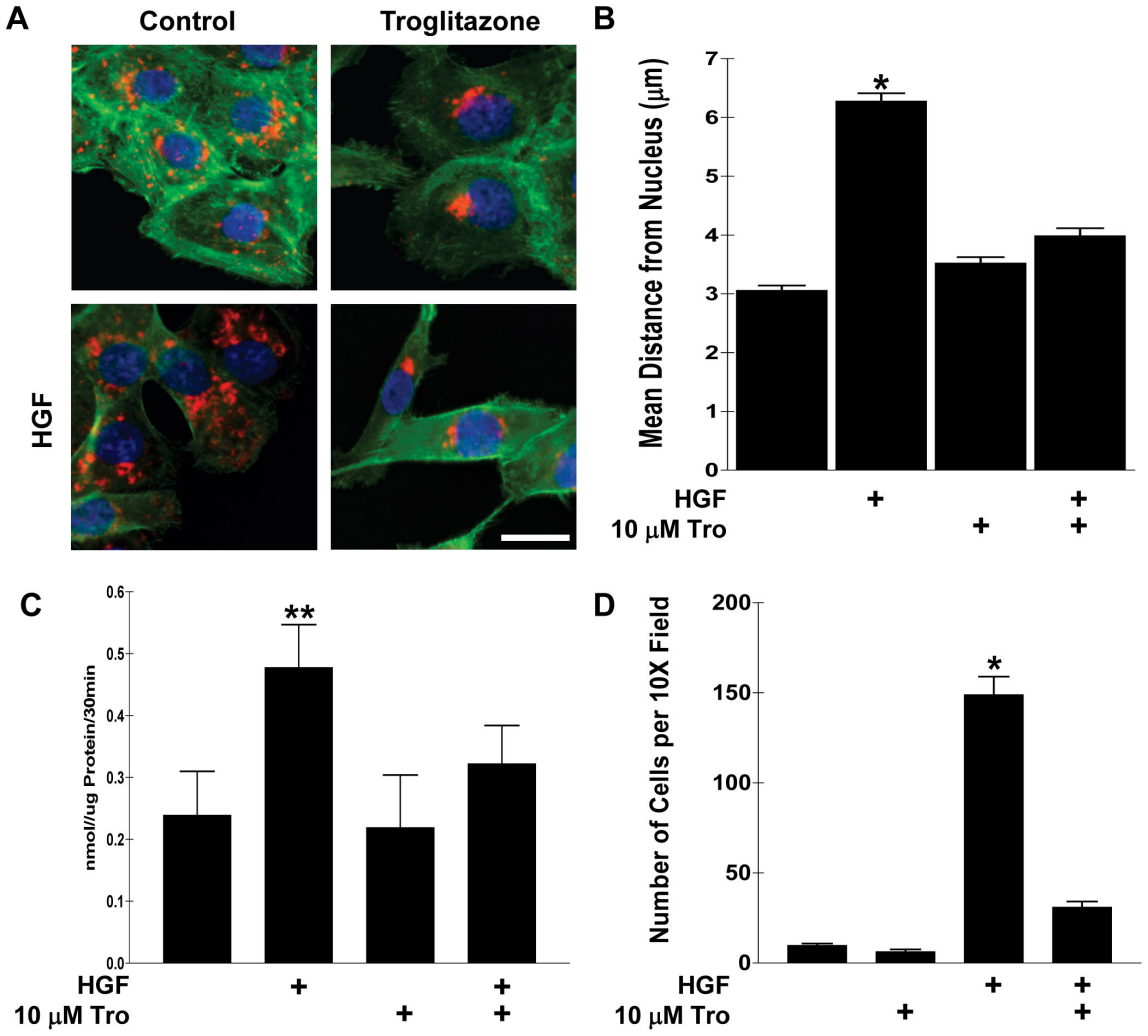
Troglitazone prevents HGF-induced cell surface-directed lysosome trafficking, cathepsin B secretion, and invasion. A) DU145 prostate tumor cells were treated with 10 µM Troglitazone and/or HGF overnight, followed by I.F. microscopy to visualize lysosomes (red), actin (green), and nuclei (blue). Troglitazone also causes lysosomes to cluster juxtanuclear to the cell nuclei. B) Quantitation of the spatial distribution of lysosomes is shown as mean distance from individual cell nuclei for each treatment condition. Error bars represent the s.e.m of 30 cells from at least three independent experiments. C) DU145 cells were treated for 16 hrs with Troglitazone and/or HGF and the amount of Cathepsin B secreted into the culture media was detected utilizing a cathepsin B activity assay (see methods and materials). D) DU145 cells were seeded onto Matrigel-coated transwell inserts and allowed to invade for 24 hrs. Treatments were added where indicated to both the top and bottom of the insert. *Statistical significance (p<0.001) versus control; **Statistical significance (p<0.01) versus control. Scale bars: 10 µm.

We have previously demonstrated that HGF can induce the cell surface-directed trafficking of lysosomes [10]. In addition, we have demonstrated that Troglitazone prevented acidic pHe-induced cell-surface directed lysosome trafficking [13]. In order to determine if Troglitazone can also prevent HGF-induced cell surface-directed lysosome trafficking, DU145 prostate tumor cells were co-treated with 10 µM Troglitazone and 33 ng/µl HGF for 16 hrs, after which lysosomes were visualized by I.F. microscopy using LAMP1 (lysosome-associated membrane protein-1) as a previously established lysosome marker [9]. Representative I.F. images shown in Figure 1A demonstrate that HGF induced the trafficking of lysosomes to the cell surface as previously published and that Troglitazone not only prevented HGF-induced cell surface-directed trafficking, but also induced the retrograde trafficking and coalescence of lysosomes adjacent to cell nuclei. Lysosomes coalesced over the microtubule-organizing center (MTOC) in Troglitazone treated cells (data not shown; [13]). Quantification of lysosome location as the distance from the cell nucleus (using previously described software [9], [22]; Figure 1B) demonstrated that HGF caused the redistribution of lysosomes toward the cell surface (away from the cell nucleus) and Troglitazone prevented HGF-induced cell surface-directed lysosome trafficking.

To demonstrate a functional consequence of lysosome trafficking, the activity of cathepsin B secreted into the culture media was measured. The increase in cathepsin B activity is directly proportional to the amount of cathepsin B secreted from the tumor cells [9]. Figure 1C demonstrates that Troglitazone significantly prevented HGF-induced cathepsin B secretion. Finally, Matrigel coated transwell inserts were utilized to perform *in vitro* invasion assays. Troglitazone significantly prevented the invasion of HGF treated cells (Figure 1D). The decrease in invasion was not due to cell death (data not shown; [10]).

### Members of the Thiazolidinedione (TZD) Family of Compounds Differentially Inhibit HGF-induced Cell Surface-directed Lysosome Trafficking and Cell Invasion

Troglitazone is a member of the Thiazolidinedione family of compounds. Therefore, we tested the ability of other TZDs to inhibit HGF-induced cell surface-directed lysosome trafficking and cell invasion. As shown in Figure S1A and quantitated in Figure S1B, Ciglitazone and Rosiglitazone also prevented cell surface directed lysosomal trafficking; whereas, Pioglitazone had no effect. The differential effects of the three TZDs on lysosome clustering were ranked: Troglitazone>Ciglitazone = Rosiglitazone. Since Pioglitazone had no effect on lysosome trafficking, we compared the effects of Troglitazone and Pioglitazone on cell invasion. Figure S1C demonstrates that Troglitazone significantly inhibited cell invasion through Matrigel coated filters; whereas, Pioglitazone had no effect on cell invasion, suggesting that the spatial location of lysosomes is an inherently important aspect in tumor cell invasion.

### The Rab7 GTPase is Necessary for the Prevention of HGF-induced Cell Surface-directed Lysosome Trafficking, Cathepsin B Secretion, and Cell Invasion

**Figure 2:**
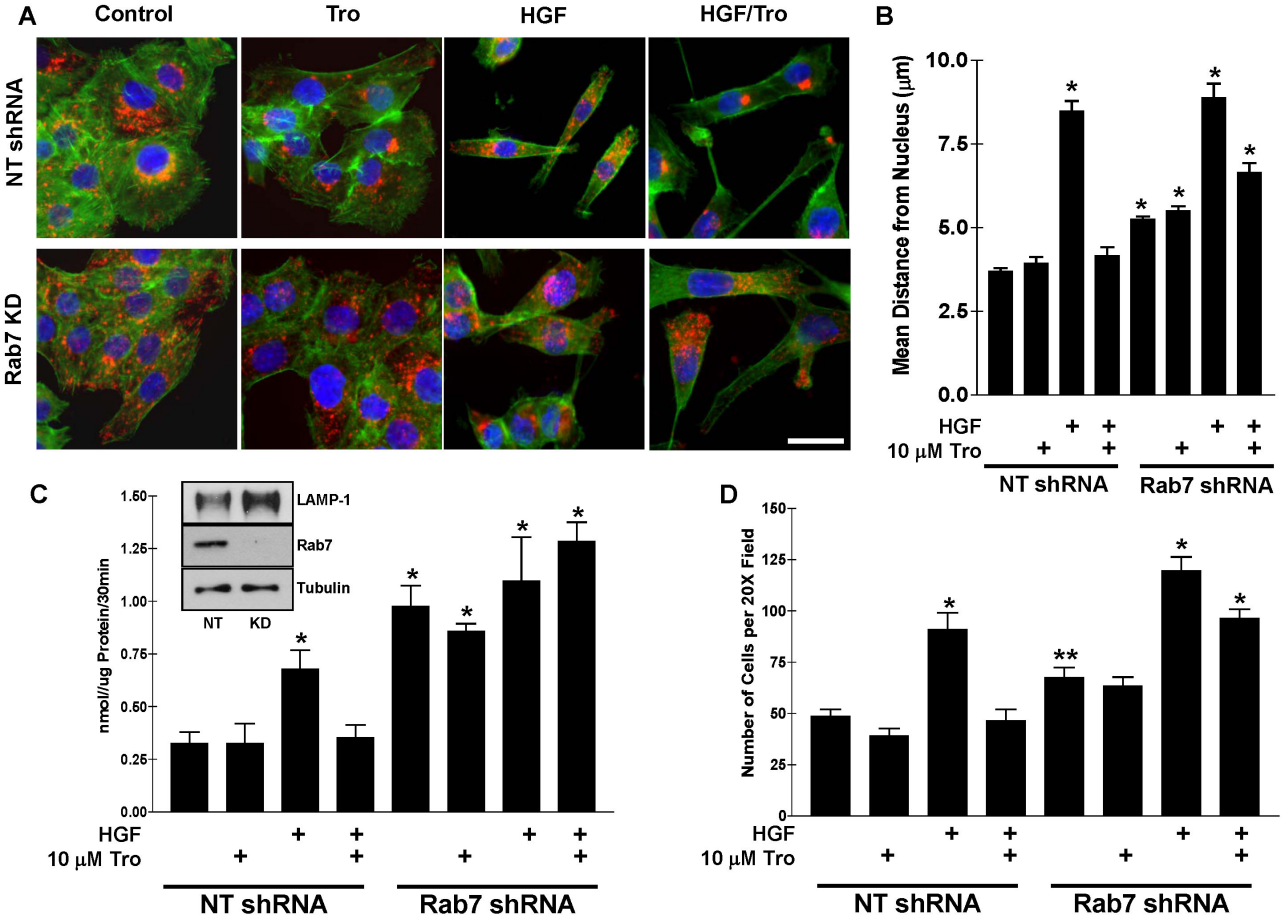
The GTPase Rab7 is necessary for Troglitazone-mediated prevention of HGF-induced cell surface-directed lysosome trafficking, cathepsin B secretion, and invasion. A) I.F. microscopy was performed on DU145 prostate tumor cells expressing either a non-target (scrambled) shRNA or a Rab7-directed shRNA to visualize lysosomes (red), actin (green), and nuclei (blue). Rab7 shRNA expression prevents Troglitazone-induced juxtanuclear lysosome clustering and causes lysosomes to traffic to the cell surface independent of HGF. B) Quantitation of the spatial distribution of lysosomes is shown as mean distance from individual cell nuclei for each treatment condition. Error bars represent the s.e.m of 30 cells from at least three independent experiments. C) NT and Rab7 shRNA expressing DU145 cells were treated for 24 hrs with Troglitazone and/or HGF and the amount of Cathepsin B secreted into the culture media was detected utilizing a cathepsin B activity assay (see methods and materials). Cathepsin B secretion is increased and Troglitazone no longer prevents secretion in the Rab7 knockdown cells. Inset indicates LAMP-1 and Rab7 expression in the nontarget (NT) or Rab7 shRNA (KD) stable cell lines by Western blot. D) NT and Rab7 shRNA expressing DU145 cells were seeded onto Matrigel-coated transwell inserts and allowed to invade for 24 hrs. Treatments indicated were added to both the top and bottom of the insert. *Statistical significance (p<0.001) versus NT control; **Statistical significance (p<0.01) versus NT control. Scale bars: 10 µm.

Although we have recently implicated Rab7 as a negative regulator of lysosome trafficking, cathepsin B secretion, and cell invasion [10], [13], we have not demonstrated the role of Rab7 in the context of Troglitazone and HGF. Therefore, DU145 cells expressing Rab7 shRNA (Rab7 knockdown was 95% compared to a non-target (NT) shRNA expressing cell line (Figure 2C inset) [10]), were treated with HGF or Troglitazone for 16 hrs. I.F. microscopy (Figure 2A) demonstrated that Rab7 was required for Troglitazone to prevent HGF-induced cell surface-directed lysosome trafficking. In addition, lysosomes in Rab7 shRNA expressing cells were found closer to the cell surface even in the absence of HGF. Quantitation of nucleus-lysosome distance (Figure 2B) shows that lysosomes are significantly further from the cell nuclei and that Troglitazone was ineffective at inducing juxtanuclear lysosome aggregation in the Rab7 shRNA expressing cells.

Similar to the distribution of lysosomes, Rab7 shRNA expressing cells demonstrated increased cathepsin B secretion in the absence of HGF (Figure 2C). Moreover, Troglitazone was unable to prevent HGF-induced cathepsin B secretion in cells expressing Rab7 shRNA. Similar results were obtained for cell invasion. Troglitazone prevented the HGF-induced increase in tumor cell invasion in NT shRNA control cells, but not in the Rab7 knockdown cells (Figure 2D). Moreover, Rab7 shRNA expressing cells were significantly more invasive compared to NT shRNA cells in the absence of any stimulus. Taken together these data suggest that i.) Rab7 expression is required for Troglitazone to prevent HGF-induced lysosome trafficking and ii.) Rab7 acts as a negative regulator of cell surface-directed lysosome trafficking, cathepsin B secretion, and tumor cell invasion.

### Rab7 shRNA Expressing Tumors Grow Larger due to Increased Proliferation and Decreased Apoptotic Rates

**Figure 3:**
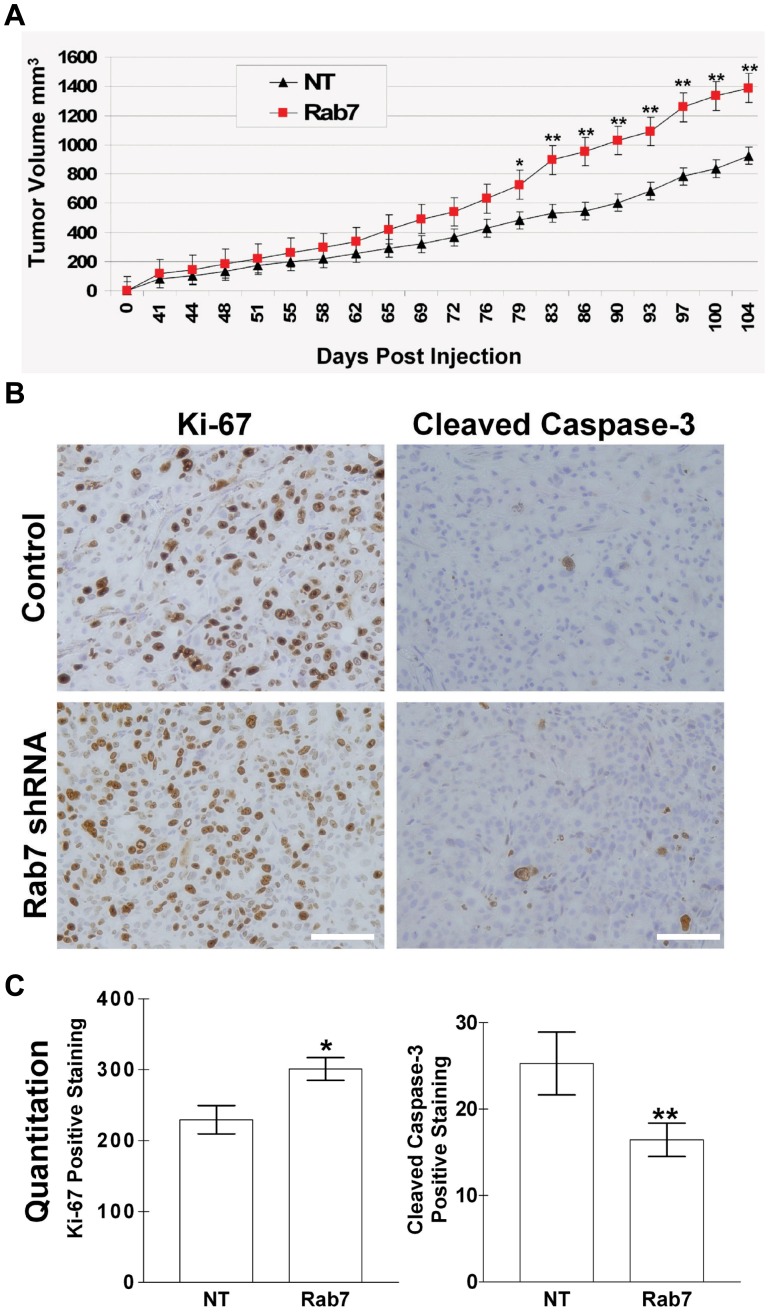
Rab7 shRNA expressing tumors grow larger, demonstrate increased cell proliferation, and decreased apoptosis *in vivo*. A) SCID/Bg mice were injected with 2×10^6^ NT shRNA (n = 6) or Rab7 shRNA (n = 7) expressing DU145 cells s.c. Tumors became measureable by day 41 and were measured via digital calipers twice per week. Mice were sacrificed once the largest tumor reached 1.4 cm^2^. Data are expressed as cubic millimeter volume. Error bars represent s.e.m. of 6 and 7 tumors respectively. *Statistical significance (p<0.05) versus NT shRNA tumors; ** Statistical significance (p<0.001) versus NT shRNA tumors. B) Immunohistochemistry was performed on formaldehyde fixed tumor tissue (see methods and materials). Cell proliferation was assessed using Ki-67 as a marker and apoptotic cells were visualized using Cleaved Caspase-3 as a marker. C) IHC was quantitated by counting Ki-67 or Cleaved Caspase-3 positive cells. Three random fields per tumor were manually counted. The number of positively staining cells were graphed. *Statistical significance (p<0.01) versus NT shRNA tumors; **Statistical significance (p<0.05) versus NT shRNA tumors. Scale bars: 100 µm.

Since Rab7 shRNA expressing DU145 cells were more invasive *in vitro*, these cells were injected sub-cutaneous into the hind flank of SCID mice and tumor volume was measured over time to determine what, if any, effect Rab7 down regulation had *in vivo*. Tumors derived from Rab7 shRNA expressing cells grew larger than those from NT shRNA expressing cells (Figure 3A). The difference became significant (p<0.05) at day 79 post injection and highly significant past 79 days (p<0.001). It is interesting to note that increased proliferation was not detected in the Rab7 shRNA expressing cell *in vitro* (Figure S2). After the largest tumor reached 1.4 cm^3^, all mice were sacrificed and tumors were surgically removed. The surrounding murine tissues adjacent to the tumors were also harvested at this time. Tumors were then divided in half and either fixed in ten percent formalin or flash-frozen.

Formalin fixed tumors were then sectioned and embedded in paraffin for immunohistochemical (IHC) analysis. Since a difference in tumor growth rate was detected, tumor sections were stained with Ki-67 (a proliferation marker) and Cleaved Caspase-3 (an apoptotic marker) and counterstained with hematoxylin. Representative staining is shown in Figure 3B. IHC staining was quantitated by manually counting Ki-67 or Cleaved Caspase-3 stained cells. Quantitation shown in Figure 3C demonstrates a significant (p<0.01) increase in Ki-67 positive staining and significant (p<0.05) decrease in Cleaved Caspase-3 positive staining, suggesting that the increase in tumor volume is due to both increased proliferation and decreased apoptotic rates.

### Rab7 shRNA Expressing Tumors Exhibit Increased Invasion and Tissue Remodeling

**Figure 4:**
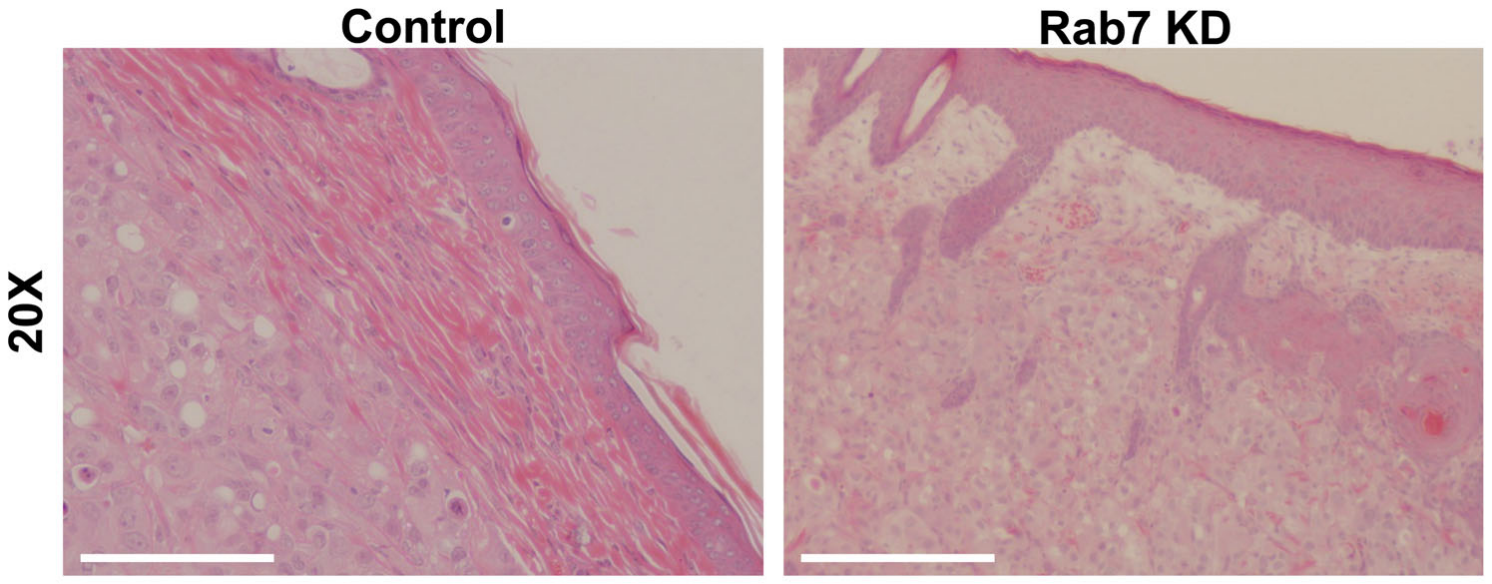
Rab7 shRNA expressing tumors demonstrate increased cell invasion into surrounding murine tissue. H&E stained s.c. tumor cross sections with the surrounding murine tissue and skin shown adjacent to the tumor. Rab7 knockdown tumors display increased infiltration into the tissue layer underlying the skin compared to control tumors. In addition, the tissue displays increased disorder and loss of integrity surrounding the Rab7 knockdown tumors. Scale bar: 100 µm.

Paraffin embedded tumor sections were stained with hematoxylin and eosin and visualized at two different magnifications. As stated above, during surgical removal of the tumors, the adjacent murine tissues and skin were left intact. Representative cross-sections of the H&E stain are shown in Figure 4. Control (NT shRNA expressing) tumors show overall tissue integrity with a thin layer of skin overlying a much thinker layer of organized, intact tissue (striated cells; indicated by the arrows). Few tumor cells can be detected invading into this tissue layer in control tumors. In contrast, the Rab7 shRNA expressing tumors displayed a much more aggressive phenotype. The integrity of the surrounding murine tissue was severely compromised and numerous tumor cells were found interspersed within the murine tissue or in most cases, the striated architecture of the surrounding tissue was entirely compromised. Therefore, we conclude that not only were the Rab7 shRNA expressing tumors larger, they had increased capacity to remodel and invade into the surrounding tissue.

### Rab7 mRNA is Down-regulated in Prostate Cancer Epithelial Cell Biopsies, but not in the Surrounding Stromal Cells, nor in BPH

Since Rab7 down-regulation increased tumor growth *in vivo* and increased invasive capacity *in vitro*, the *ONCOMINE* database was searched to determine the differential mRNA levels of Rab7 in human prostate cancer. Analysis of the differential regulation of Rab7 in prostate cancer revealed six datasets spanning nine different prostate tissue subsets. As seen in Table 1, several studies showed a significant fold decrease in Rab7 mRNA expression ranging from −2.501 to −1.128; however, two studies were equivocal with one probe detecting an increase and a second probe detecting a decrease of Rab7 mRNA in prostate tumor samples, and one study did not detect any change in Rab7 mRNA expression. Furthermore, an analysis of PIN lesions also showed a significant −1.939 fold decrease in Rab7, whereas two separate analyses of benign prostatic hyperplasia (BPH) failed to show a reduction in Rab7. Taken together, a trend appears whereby Rab7 is down-regulated early in prostate tumor development (PIN lesions) and remains down-regulated during tumor progression, but is not down-regulated in benign conditions such as BPH.

**Table 1:** Differential regulation of Rab7 in tumor biopsy specimens by microarray analysis.

Differential Rab7 Expression Levels Reported in the *ONCOMINE* Database (Prostate Cancer)				
Type of Analysis	Fold Change	*P* value	Reference	Sample Notes
PCa vs. Normal Prostate	−2.501	<0.0001	[41]	Samples contained 90% cancerous epithelial cells
PIN Epithelia vs. Normal Prostate	−1.939	0.013	[40]	Enriched for PIN epithelial cells
PCa Epithelia vs. Normal Prostate	−1.743	0.013	[40]	Enriched for cancerous epithelial cells
PCa vs. Normal Prostate	−1.265	<0.001	[42]	Pooled tumor samples–contained stromal cells
PCa vs. Normal Prostate	−1.118	0.003	[43]	Stromal cells included (not enriched for epithelial cells)
PCa vs. Normal Prostate	1.285	0.016	[43]	Stromal cells included (not enriched for epithelial cells)
PCa vs. Normal Prostate	−1.128	0.024	[44]	Stromal cells included (not enriched for epithelial cells)
PCa vs. Normal Prostate	1.294	0.004	[44]	Stromal cells included (not enriched for epithelial cells)
PCa vs. Normal Prostate	−1.012	0.32	[45]	Stromal cells included (not enriched for epithelial cells)
BPH Epithelia vs. Normal Prostate	−1.165	0.35	[40]	Benign epithelia
BPH Stroma vs. Normal Prostate	−1.118	0.039	[40]	Benign stroma

The *ONCOMINE* database was searched for differential expression of Rab7A mRNA in prostate cancer.

### Rab7 Knockdown Causes an Increase in c-Met Levels

**Figure 5:**
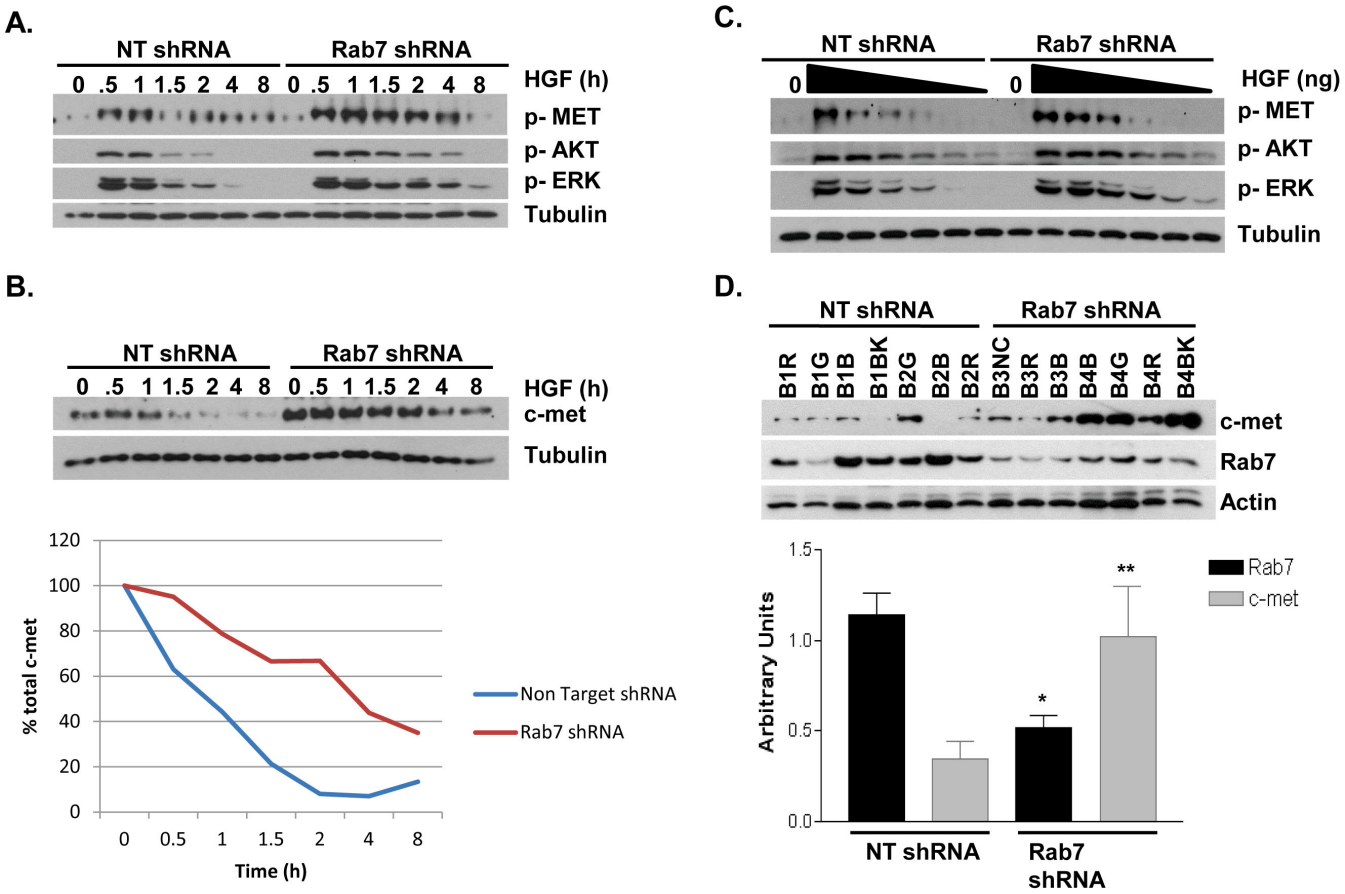
Reduced Rab7 expression results in increased c-met expression and enhanced signaling. A) Nontarget (NT) vector control or Rab7 shRNA expressing DU145 cells were exposed to HGF for the indicated times. Whole cell lysates were collected and c-met, Akt, and Erk phosphorylation was detected by Western blotting. B) Cells were treated with HGF for the indictated time periods and levels of total c-Met protein were determined by Western blot analysis. Densitometry from three independent experiments was used for graphical representation of c-Met loss over time. C) Cells were treated with the indicated concentrations of HGF for 30 minutes followed by Western blot analysis of the indicated proteins. D) Protein lysates of xenograft tumors were harvested and levels of total c-met and Rab7 protein were detected by Western blot. The bar graph represents the average c-met and Rab7 expression of seven tumors per treatment group as determined by densitometry of Western blot. * = p<0.0007 compared to NT shRNA Rab7. ** = p<0.03 compared to NT shRNA c-met. Error bar represent SEM.

To begin to determine the mechanisms regulating an increase in tumor proliferation and invasion in cells containing reduced Rab7, we examined the levels and activation of c-Met in vector control and Rab7 shRNA expressing cells. The addition of HGF to control cells resulted in increased c-Met phosphorylation and activation of downstream signaling pathways followed by a gradual decrease over the next few hours. In contrast, the addition of HGF to Rab7 knockdown cells resulted in a more robust increase in c-Met phosphorylation and a slower loss of activated Met and downstream signaling partners over time (Figure 5A). Consistent with these data, analysis of total c-Met protein in control and Rab7 knockdown cells indicated that c-Met levels were higher in cells with less Rab7 and that the HGF-mediated loss of c-Met was also attenuated (Figure 5B).

Since c-Met levels were higher in Rab7 knockdown cells, we predicted that these tumor cells would be more responsive to lower concentrations of HGF. As indicated in Figure 5C, Rab7 knockdown cells responded to lower concentrations of HGF as measured by activation of c-Met, Akt and Erk.

Finally, to determine if c-Met levels were decreased in Rab7 knockdown mouse xenografts, protein from frozen xenograft tumor tissue was harvested and prepared for Western blot analysis. Figure 5D indicates that tumors formed from vector control DU145 cells on average contained more Rab7 and less c-Met than tumors formed from the Rab7 knockdown cells. This demonstrates that the Rab7 knockdown phenotype is maintained during tumor growth and that the concurrent overexpression of c-Met remains stable in tumors.

## Discussion

Prostate cancer is a clinically heterogeneous disease, ranging from relatively indolent primary tumors to lethal metastatic disease. Mortality rates of late stage, metastatic prostate cancer remain high and there is no definitive treatment. Therefore, understanding the molecular genetics and biology of aggressive prostate cancer is urgently needed in order to identify new drug targets and biomarkers. In previous studies, we explored the atypical trafficking of lysosomes in tumors of various tissue origin [9]. Noteworthy, lysosomes are being considered as a valuable target for cancer therapeutics [23]. We and others have shown increased cathepsin B secretion and tumor cell invasion in response to factors within the tumor microenvironment including acidic pHe and HGF [7], [8], [9], [10], [11], [13], [24]. In fact, our conclusion that lysosome distribution can regulate cell invasion is supported by studies in invasive brain cancer [25], [26]. In this report, we demonstrate for the first time that Troglitazone and other members of the Thiazolidinedione family differentially prevent HGF-induced lysosome trafficking. In addition, Rab7 down-regulation increased tumor cell invasion and prevented the action of Troglitazone. Thus, taken together these studies suggest that NHE inhibitors such as EIPA [9], [10] and now Troglitazone prevent HGF- and acidic pHe-induced lysosome trafficking, which requires the GTPase Rab7. It could be argued that Troglitazone is acting via multiple mechanisms to prevent cell invasion in addition to the effects on lysosome trafficking, as Troglitazone also activates the transcription factor PPAR-γ, and the MAPK and PI3K signaling pathways [13], [27], [28]. For this reason, we focused the rest of the study on the role of Rab7 in tumorigenesis, independent of Troglitazone or other Thiazolidinediones.

Herein we report that Rab7 is a negative regulator of cell-surface directed lysosome trafficking and cathepsin B secretion, and reduction of Rab7 protein results in increased cell invasion. Furthermore, this is the first report to our knowledge demonstrating that tumors derived from Rab7 shRNA expressing cells grew faster and exhibited an increased invasive phenotype *in vivo*. This result was unexpected as the proliferation rate of Rab7 shRNA expressing cells *in vitro* is unchanged compared to vector control cells (Figure S2). In addition to increased proliferation and decreased apoptosis *in vivo,* H/E staining detected an increase in tumor cell invasion into the adjacent murine tissue (Figure 4) from tumors derived from Rab7 shRNA expressing cells. However, upon tumor dissection, we did not visually detect overt invasion into the abdominal wall, thus further studies using other mouse models of metastasis or orthotopic models are needed to further define the role of Rab7 in tumor cell invasion and metastasis.

One possible mechanism for the increased proliferation and decreased apoptotic rates seen *in vivo* is the regulation of growth factor receptors through endocytosis, which is often deregulated in cancer [29]. Rab7 is a key player in the down regulation of growth factor-induced signaling by regulating endocytic trafficking of internalized receptor tyrosine kinases, including c-Met, whereby Rab7 regulates the fusion of late endosomes to lysosomes, inducing the degradation of the receptor, which is essential for regulation of growth factor receptor signaling [30]. Thus, Rab7 could be considered a negative regulator of many pro-survival signals from the cell surface. In fact, Sakane *et al.* [31] have shown that the Rab7 effector Rabring7 (Rab7-interacting RING-finger protein) is an E3 ligase involved in epidermal growth factor receptor (EGFR) degradation.

Interestingly, we observed that total c-Met protein levels were increased in cells with reduced Rab7 expression, a phenotype that was maintained throughout xenograft tumor growth (Figure5). Rab7 is known to mediate endosome/lysosome fusion, a process that is necessary for the normal turnover and degradation of many proteins, including receptor tyrosine kinases [29]. A reduction in Rab7 protein may possibly slow the basal degradation rate of c-Met, thus increasing the half life of the protein and allowing for accumulation of c-Met in tumor cells. In support of this mechanism, we found that total c-Met protein was degraded more slowly in response to HGF stimulation in cells expressing Rab7 shRNA when compared to the NT shRNA control, indicating that Rab7 is indeed controlling c-Met degradation in our system. Additionally, deregulated c-Met endosomal trafficking in the Rab7 knockdown cells may also contribute to prolonged signaling and increased sensitivity to HGF. By disrupting normal c-Met endosomal/lysosomal trafficking via depletion of Rab7, c-Met may be maintained in the endosome allowing for sustained signaling and/or recycled back to the plasma membrane resulting in increased signaling by those c-Met receptors that would normally be targeted for degradation. In support of a role for Rab7 regulating c-Met, Rab7 is also known to control the trafficking and regulate the signaling of other receptor tyrosine kinases in a variety of cell systems, including the oncogenic EGFR [32], [33], [34], [35], [36]. Thus, the increased invasion and proliferation observed in the Rab7 knockdown cells *in vivo* (Figures 3 and 4) may not be solely due to peripheral lysosome trafficking, but may also be the result of increased c-Met expression and activity. It is interesting to note that Rab7 shRNA expressing cells proliferate and undergo apoptosis at rates similar to NT shRNA control cells *in vitro*, but proliferate more rapidly *in vivo* (Figure S2 and Figure 3). This discrepancy may be attributed to c-Met activation from murine HGF *in vivo*, whereas HGF was not added to determine the proliferation rates of Rab7 knockdown cells *in vitro*. Thus, not only does Rab7 knockdown increase invasion by promoting anterograde lysosome trafficking and protease secretion, this phenotype also results in higher levels of c-Met, prolonged signaling, and an increased sensitivity to HGF that may contribute to pro-survival and pro-invasive signals in tumor cells.

In support of this mechanism, the Edinger group [19], [37] has demonstrated that Rab7 is activated and induces apoptosis (involving PKCδ) under conditions of growth factor withdrawal, suggesting that Rab7 activation limits growth factor-independent cell survival. Put another way, Rab7 is pro-apoptotic thus; the down-regulation or inactivation of Rab7 supports cell proliferation and survival. The complete underlying mechanisms of Rab7 in cell survival, apoptosis, and tumorigenesis are not yet completely understood, and this remains an active area of study. Furthermore, modulation of Rab7 activity may also be of benefit in other research areas including infectious diseases, osteoporosis, and autophagy. The role of Rab7 in these processes has recently been reviewed [17].

Although, Rab7 has been reported to be overexpressed in diffuse malignant peritoneal mesothelioma and autonomous thyroid adenomas [38], [39], we searched the *ONCOMINE* database to determine the levels of Rab7 mRNA in prostate tumor biopsies (Table 1). Taken together the analysis suggests a trend by which Rab7 mRNA levels are decreased in the transition from normal prostatic epithelium to PIN and remains low in prostate adenocarcinoma. It is important to note that several datasets did not reflect significant down regulation of Rab7 in prostate tumors or that the mRNA levels were equivocal. As human prostate biopsy samples contain both tumorigenic epithelial cells and the surrounding stromal cells, it is interesting to note that the tumor samples in the studies demonstrating a reduction in Rab7 were enriched for high epithelial cell content (Table 1). Therefore, since the studies demonstrating low or non-significant Rab7 down-regulation also contained stromal cells in the analysis, it is tempting to speculate that the presence of stromal cells in the analysis may actually mask the potential down-regulation of Rab7; contributing to false negative results in the datasets. In fact, prostate epithelia and stromal cells are known to have different gene signatures [40]. None-the-less, the fact that prostate carcinoma and PIN tissue demonstrate decreased Rab7 mRNA, but not the BPH samples suggests that Rab7 be commonly down regulated during prostate tumor progression. It is of note that a single prostate cancer cell line was utilized for the work in this research as it was ideal for our *in vivo* model; however, the influence of Rab7 on lysosome transport has been documented in numerous cell lines. This, along with the correlations identified in the oncomine analysis, suggest the results herein will be expanded to numerous epithelial cancer models.

In conclusion, our results add to the increasing evidence that Rab7 functions as a prostate tumor suppressor and raises the interesting possibility that drugs which promote Rab7 activity may be possible cancer therapeutics. It is tempting to speculate that members of the thiazolidinediones may activate Rab7 and serve as parent compounds for medicinal chemists to modify, creating less toxic derivatives that increase Rab7 activity.

## Supporting Information

Figure S1 **Members of the Thiazolidinedione family differentially inhibit HGF-induced cell surface-directed lysosome trafficking and induce JLA.** Troglitazone, Ciglitazone, and Rosiglitazone inhibit HGF-induced cell surface-directed lysosome trafficking; whereas, Pioglitazone does not affect lysosomal trafficking. A) I.F. microscopy indicates the effects of Troglitazone, Ciglitazone, Rosiglitazone, and Pioglitazone (all at 10 µM) on the spatial distribution of lysosomes (red) in DU145 cells. Actin (green) and nuclei (blue) are also shown. B) Quantitation of the spatial distribution of lysosomes is shown as mean distance from individual cell nuclei for each treatment condition. Error bars represent the s.e.m of 30 cells from at least three independent experiments. C) DU145 cells were seeded onto Matrigel-coated transwell inserts and allowed to invade for 24 hrs. HGF and the various Thiazolidinediones were added where indicated to both the top and bottom of the insert. *Statistical significance (p<0.001) versus control; **Statistical significance (p<0.01) versus control. Scale bars: 10 µm.
(TIF)

Figure S2 **Rab7 shRNA expression does not effect in vitro proliferation or apoptosis.** DU145 cells expressing either NT or Rab7 shRNA were cultured in 96-well plates. A.) Cell viability was assessed over time utilizing an MTS assay (see methods and materials for details). Error bars represent the s.e.m of 8 replicates. B, C) Cells were plated at 30% confluence in a 96 well plate and treated with 5 µM CellPlayer™ Kinetic Caspase-3/7 Apoptosis reagent (Essen ) in the presence of complete media. Cells were grown for 48 hours and phase contrast and fluorescent images were acquired in real time every 4 hours for the duration of the experiment using the IncuCyte Zoom imaging platform (Essen ). B) Graphical representation of the green confluence for each cell line over time. Error bars represent SEM. C) Representative images of Rab7 KD and Non Target shRNA expressing cells at T0 and T48. Green represents cells that have activated caspase -3/7 as readout for apoptosis.
(TIFF)
