# Supplementary figures and images for: Supporting a Role for the GTPase Rab7 in Prostate Cancer Progression
Source: PLoS One. 2014 Feb 5;9(2):e87882. doi: 10.1371/journal.pone.0087882 (PMC3914878; doi:10.1371/journal.pone.0087882)

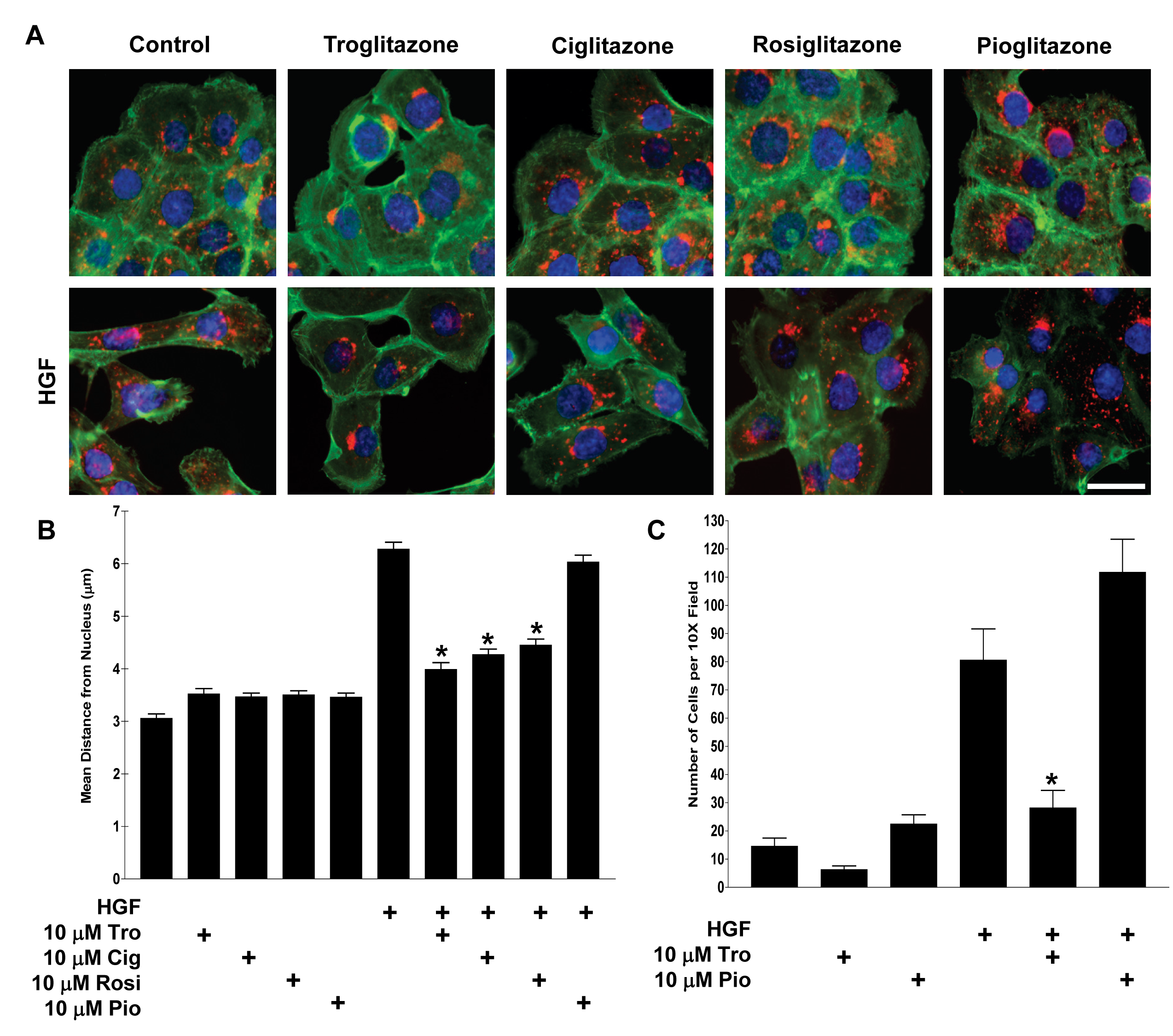

Supplement: Figure S1 — Members of the Thiazolidinedione family differentially inhibit HGF-induced cell surface-directed lysosome trafficking and induce JLA. Troglitazone, Ciglitazone, and Rosiglitazone inhibit HGF-induced cell surface-directed lysosome trafficking; whereas, Pioglitazone does not affect lysosomal trafficking. A) I.F. microscopy indicates the effects of Troglitazone, Ciglitazone, Rosiglitazone, and Pioglitazone (all at 10 µM) on the spatial distribution of lysosomes (red) in DU145 cells. Actin (green) and nuclei (blue) are also shown. B) Quantitation of the spatial distribution of lysosomes is shown as mean distance from individual cell nuclei for each treatment condition. Error bars represent the s.e.m of 30 cells from at least three independent experiments. C) DU145 cells were seeded onto Matrigel-coated transwell inserts and allowed to invade for 24 hrs. HGF and the various Thiazolidinediones were added where indicated to both the top and bottom of the insert. *Statistical significance (p<0.001) versus control; **Statistical significance (p<0.01) versus control. Scale bars: 10 µm. (TIF) [file pone.0087882.s001.tif]

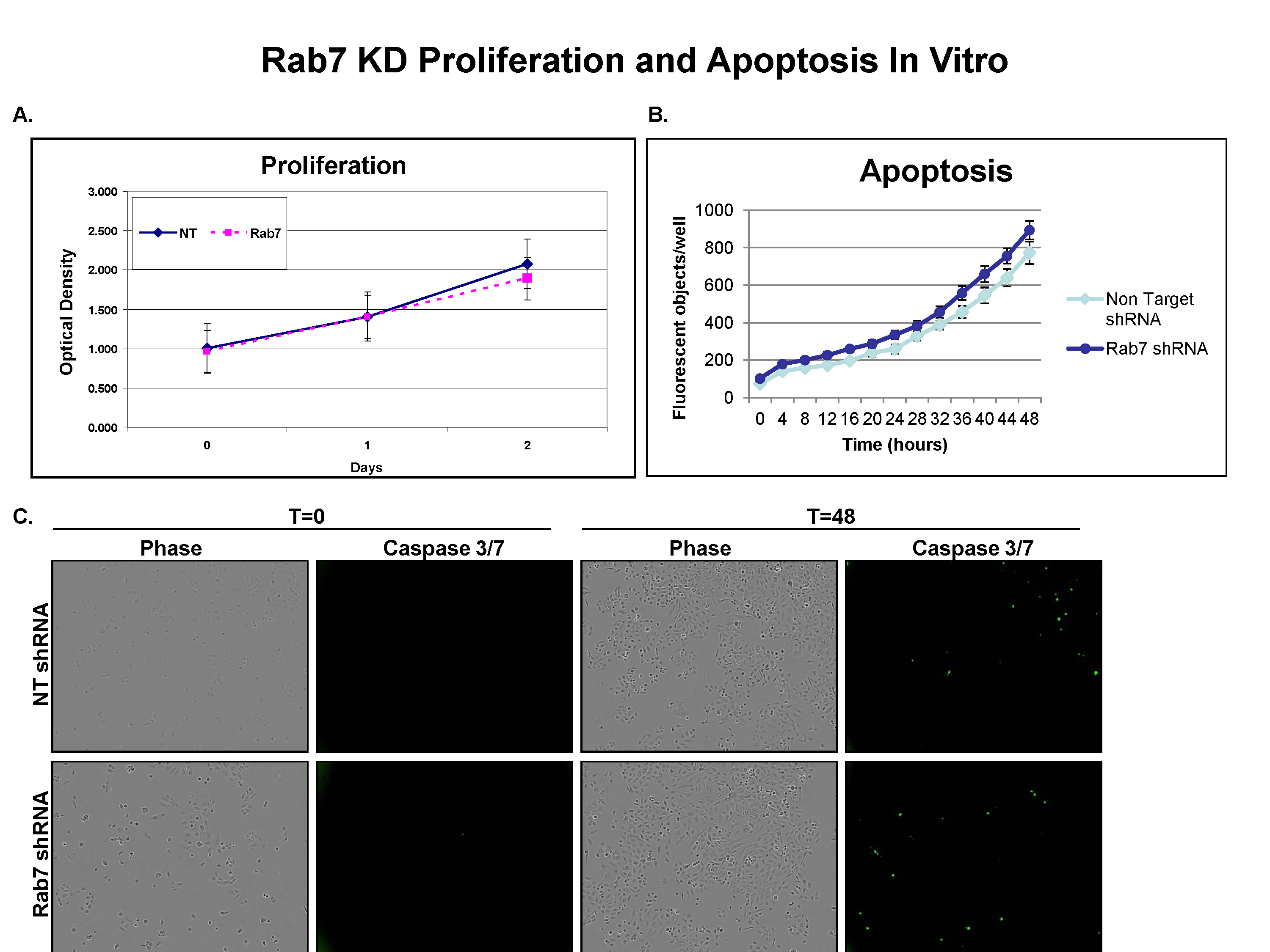

Supplement: Figure S2 — Rab7 shRNA expression does not effect in vitro proliferation or apoptosis. DU145 cells expressing either NT or Rab7 shRNA were cultured in 96-well plates. A.) Cell viability was assessed over time utilizing an MTS assay (see methods and materials for details). Error bars represent the s.e.m of 8 replicates. B, C) Cells were plated at 30% confluence in a 96 well plate and treated with 5 µM CellPlayer™ Kinetic Caspase-3/7 Apoptosis reagent (Essen ) in the presence of complete media. Cells were grown for 48 hours and phase contrast and fluorescent images were acquired in real time every 4 hours for the duration of the experiment using the IncuCyte Zoom imaging platform (Essen ). B) Graphical representation of the green confluence for each cell line over time. Error bars represent SEM. C) Representative images of Rab7 KD and Non Target shRNA expressing cells at T0 and T48. Green represents cells that have activated caspase -3/7 as readout for apoptosis. (TIFF) [file pone.0087882.s002.tiff]
